# Direct and Latent Effects of Pathogen Exposure Across Native and Invasive Amphibian Life Stages

**DOI:** 10.3389/fvets.2021.732993

**Published:** 2021-10-29

**Authors:** Jenny Urbina, Evan M. Bredeweg, Andrew R. Blaustein, Tiffany S. Garcia

**Affiliations:** ^1^Environmental Sciences Graduate Program, Oregon State University, Corvallis, OR, United States; ^2^Department of Fisheries, Wildlife and Conservation Sciences, Oregon State University, Corvallis, OR, United States; ^3^Department of Integrative Biology, Oregon State University, Corvallis, OR, United States

**Keywords:** pathogen exposure, development, carry-over, embryos, larvae

## Abstract

Emerging infectious diseases are one of the multiple factors contributing to the current “biodiversity crisis”. As part of the worldwide biodiversity crisis, amphibian populations are declining globally. Chytridiomycosis, an emerging infectious disease, caused by the fungal pathogen *Batrachochytrium dendrobatidis* (*Bd*), is a major cause of amphibian population declines. This fungus primarily affects keratinized structures in larval, juvenile, and adult amphibians as well as heart function. However, we know little about how *Bd* can impact embryos as well as potential latent effects of *Bd* exposure over ontogeny. Using two different *Bd* strains and multiple exposure times, we examined the effects of *Bd* exposure in Pacific chorus frog (*Pseudacris regilla*), Western toad (*Anaxyrus boreas*) and American bullfrog (*Lithobates catesbeianus*) life stages. Using a factorial experimental design, embryos of these three species were exposed to *Bd* at early and late embryonic stages, with some individuals re-exposed after hatching. Embryonic *Bd* exposure resulted in differential survival as a function of host species, *Bd* strain and timing of exposure. *P. regilla* experienced embryonic mortality when exposed during later developmental stages to one *Bd* strain. There were no differences across the treatments in embryonic mortality of *A. boreas* and embryonic mortality of *L. catesbeianus* occurred in all *Bd* exposure treatments. We detected latent effects in *A. boreas* and *L. catesbeianus* larvae, as mortality increased when individuals had been exposed to any of the *Bd* strains during the embryonic stage. We also detected direct effects on larval mortality in all three anuran species as a function of *Bd* strain, and when individuals were double exposed (late in the embryonic stage and again as larvae). Our results suggest that exposure to *Bd* can directly affect embryo survival and has direct and latent effects on larvae survival of both native and invasive species. However, these impacts were highly context dependent, with timing of exposure and *Bd* strain influencing the severity of the effects.

## Introduction

In many organisms, exposure to stressors during embryonic or prenatal stages can result in both direct and latent effects on subsequent developmental stages. For example, in birds, conditions experienced at early life are determinants of fitness in adults (1); in snapping turtles (*Chelydra serpentina*), hatchling from eggs incubated on a wet substrate have an improved locomotor performance in comparison to hatchlings from drier substrates (2) and in fishes, embryos of pink salmon (*Oncorhynchus gorbuscha*) exposed to oil have a reduction in juvenile growth and survival (3). In amphibians, these effects can be on individual growth rates, behavior, locomotion or immunology (4–7). Thus, exposure to predator cues in the pinewoods tree frog *Hyla femoralis* slowed larval growth and development, resulting in metamorphs with relatively smaller body sizes (8). In amphibians, repeated exposure at early life stages to other environmental stressors, such as contaminants, predator cues, and pathogens, can produce latent effects in juvenile and adult amphibians (4, 9, 10). Particularly in amphibian embryos, environmental cues can cause significant changes in hatching traits (11–14). Time of hatching can change in response to risks and opportunities. For example, embryos can hatch early to escape predators and pathogens, and this life history shift can have effects that persist through later life stages (15–17). Additionally, embryos infected with water molds can suffer differential mortality rates relative to the timing of exposure to the pathogen (18). As such, the timing of pathogen exposure might play a critical role on host susceptibility to infection (19).

Host ontogeny is a key factor for examining or predicting disease dynamics. There is precedence in different systems than minimum changes in the history of exposure to pathogens during a particular developmental stage can drastically change host life history. Changes in individual susceptibility to pathogens occur throughout ontogeny in many organisms, including plants (20), insects (21), birds (22), reptiles (23), mammals (24) and amphibians (25, 26). The key, however, to understanding temporal association between pathogens and susceptibility is to empirically discern latent and direct effects within and across life history stages. We posit that amphibians can be model systems for testing these questions as they are a taxon of conservation concern, have complex life histories, and are susceptible to multiple emerging infectious diseases.

One of the most researched amphibian pathogens is the fungus *Batrachochytrium dendrobatidis* (*Bd*), which has been implicated in the decline of numerous amphibian species worldwide (27–29). Differential susceptibility to *Bd* has been documented across species (30–35), populations (36, 37), life stages (38–42), and *Bd* strains (29, 33, 43–46). However, how exposure to *Bd* in one developmental stage can produce latent affects in a later life stage is unclear. Information regarding direct *Bd* impacts on embryos is also lacking as *Bd* mainly affects keratinized structures, which are absent in embryos. Further, the importance of evolutionary relationships between *Bd* strain and the embryonic host may also have significant implications.

We explored the direct and latent effects of *Bd* exposure on both the embryonic and larval stages using three amphibian species with differential susceptibility to native and invasive *Bd* strains (33, 34, 46, 47). We posit that amphibian embryos will be susceptible to the chytrid fungus as *Bd* can produce enzymes that can destroy tissue (48–50). Further, the release of fungal toxin (30, 51) could impact embryos by delaying growth, triggering key transitions resulting in ontogenetic shifts or latent effects on life history trajectories. Finally, we hypothesized that *Bd* can impact embryos by depletion of oxygen. The lack of dissolved oxygen slows down the development in *Bufo bufo* (52) and hypoxia can kill early life stages (13). Direct or latent effects may also vary with *Bd* strain and with host species, therefore evaluating different strains is critical to disentangle intrinsic aspects of the pathogen as virulence and how it changes among hosts. We also examined the influence of *Bd* exposure on larval survival predicting that repeated exposure to *Bd* across the embryonic/larval transition would result in decreased survival.

## Materials and Methods

We studied three anuran species found in the US Pacific Northwest (PNW). These are the Pacific chorus frog (*Pseudacris regilla*) a common species throughout its PNW range found in a variety of habitats from sea level to montane regions, the Western toad (*Anaxyrus boreas*) whose populations have experienced declines across much of its historic range and American bullfrogs (*Lithobates catesbeianus*) an introduced species in the PNW (53–55). Twenty clutches of *P. regilla* were collected from Little Three creeks on 19 June 2014 (44°06′03.5″ N, 121°38′34.7″ WGS84 Deschutes County, OR, elevation = 2,000 m) and 600 eggs of *A. boreas* were collected from 20 different egg masses at Todd Lake (44°01′44.5″ N, 121°41′07.6″W WGS84 Deschutes County, OR, elevation = 1,870 m) on 29 May 2015. We collected 600 newly laid eggs from six distinct *L. catesbeianus* egg masses from William L. Finley National Wildlife Refuge on 20 May 2014 (44°25′23.6″ N, 123°18′41.8″W WGS84 Benton County, OR, elevation = 276 m). After collection, eggs were immediately transported to a climate-controlled environment at Oregon State University and held under constant temperature (14°–15.5°C) and photoperiod (12L: 12D) conditions. Less than 6 h after arrival, every clutch of *P. regilla* or group of eggs of *A. boreas* and *L. catesbeianus* were divided into three groups and each group for *P. regilla* and *A. boreas* contained ~10 eggs (±1.95 eggs), and 20 eggs for *L. catesbeianus*.

### Pre-hatch Exposure Regime

*Bd* exposure treatments were administered in either the early embryonic developmental stages or closer to hatching. Early exposure (early) corresponded to the late gastrula stages, or Gosner Developmental Stage 12 (56) while closer to hatching exposure (late) corresponded to embryos capable of muscular response, or Gosner Developmental Stage 18 (56). *Bd* strains (i.e., the isolate of the fungus used for the inoculation) included an endemic *Bd* strain (JEL 630, hereafter “West,” isolated from *L. catesbeianus* in Oregon), and a novel *Bd* strain to Oregon freshwater habitats (JEL 627, hereafter “East,” isolated from *L. catesbeianus* in Maine USA). These strains were identified as part of the North American clade (*Bd*-GPL-1) (57). We obtained cryogenically preserved culture plates from J. Longcore to prepare 1% sterile tryptone—agar media plates with 0.5 ml of stock *Bd* broth coming from each particular strain of the fungus (31). *Bd* cultures were allowed to grow for 5–8 days at 20°C before used in the experiment (31).

Using a hemocytometer, we quantified the zoospores from a pooled inoculation broth (8–12 plates per *Bd* strain). Five ml inoculations of the zoospore broth (30 K zoospores/ml) were then administered to experimental units (18 cm H × 10 cm OD high-density polyethylene graduated beakers) containing 800 ml of dechlorinated water. A similar dose was previously tested in larvae of *P. regilla* (47), *A. boreas* (33, 58, 59), and *L. catesbeianus* (32, 60). Controls were inoculated with a sham inoculum created by rinsing the same number of sterile agar plates with 5 ml of dechlorinated water.

**Figure 1:**
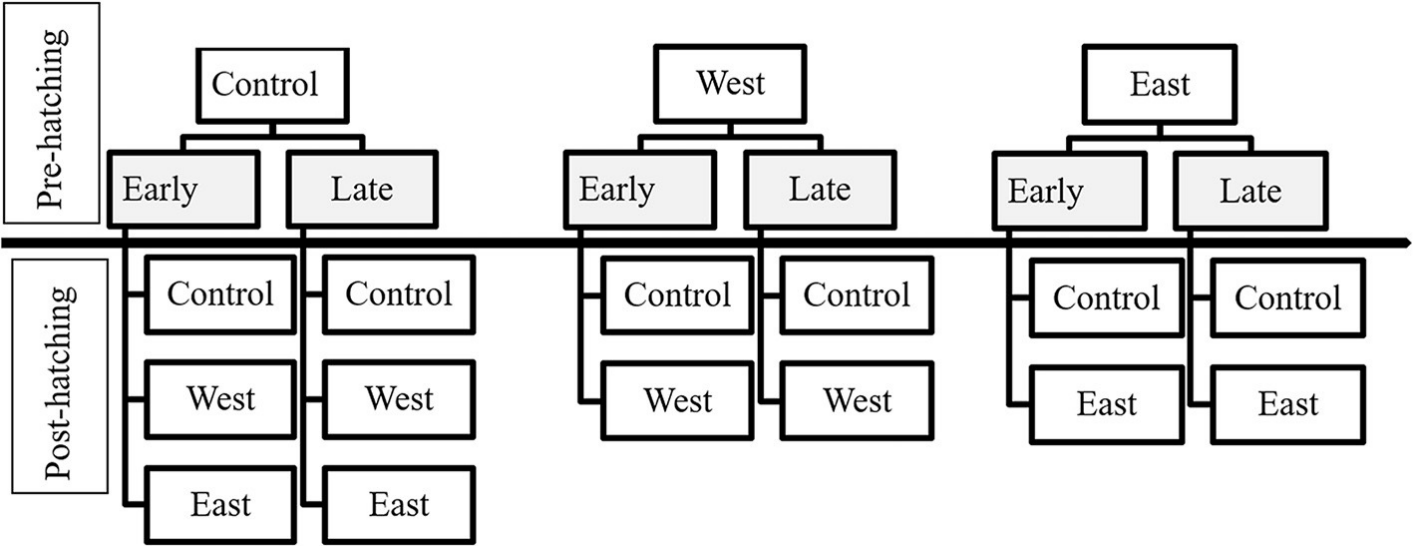
Exposure treatments of egg masses and tadpoles according to the time of exposure and chytrid strain. Pre-hatching treatments are indicated in gray and above the separation line. Treatments for tadpoles (post-hatching) are listed below the separation line.

Using a factorial experimental design, each group of eggs was assigned to a time of exposure treatment (early, late) and a *Bd* strain treatment (West, East, Control) (Figure 1, Pre-Hatching). Sixty experimental units (581 total eggs) were assigned for *P. regilla* (10 replicates per Early and Late treatment groups), 51 experimental units (506 total eggs) for *A. boreas* (8 replicates per Early exposure treatment, 9 replicates per Late exposure treatment), and 30 experimental units (600 total eggs) for *L. catesbeianus* (5 replicates for all groups, except for the East and West/Late exposure groups, which had 6 and 4 replicates, respectively) (Table 1). The length of the pre-hatching phase varied by species, lasting 19 days for *P. regilla* and *A. boreas*, and 22 days for *L. catesbeianus*. Embryos that died were preserved individually in 2.0 ml Eppendorf tubes with 95 % ethanol. No water changes were performed during the pre-hatching phase as movement associated with water changes can induce hatching, thus influencing our results. Upon hatching at Gosner stage 21, water changes were conducted weekly. We quantified the time of hatching by direct observation, and hatchling events and survival were recorded twice per day.

**Table 1:** Number of replicates per treatment per species followed by total number of eggs per treatment between parentheses.

	**Pre-hatch exposure regime**					
	***Bd*** **strain** **×** **Time exposure treatments**					
	**Control**		**East**		**West**	
**Host species**	**Early**	**Late**	**Early**	**Late**	**Early**	**Late**
*Pseudacris regilla*	10 (101)	10 (96)	10 (97)	10 (94)	10 (98)	10 (95)
*Anaxyrus boreas*	8 (80)	9 (85)	8 (84)	9 (86)	8 (84)	9 (87)
*Lithobates catesbeianus*	5 (100)	5 (100)	5 (100)	6 (120)	5 (100)	4 (80)

To analyze differences in hatching time, we compared proportions between treatments of eggs being exposed to *Bd* and control treatments (no exposure to the pathogen) using quasibinomial generalized linear models (GLM) performed independently per species. All analyses were run in R (61). To evaluate differences among strains and controls we calculated pairwise comparison using a Tukey HSD test.

### Post-hatch Exposure Regime

Upon hatching, survivors were pooled within pre-hatch treatment groups (Early or Late; East, West, Control) to standardize sample sizes for the post-hatch phase of the experiment. In this phase, larvae were either re-exposed to the same pre-hatch *Bd* strain or held as controls to estimate latent effects (Figure 1, Post-Hatch). For *P. regilla*, we had a total of 328 surviving hatchlings distributed across the larval exposure treatments for a total of 82 experimental units, resulting in 33 control replicates, 21 East strain replicates, and 28 West strain replicates. Less than 10% of eggs hatched from the East/Late pre-hatch exposure treatment group; as such, there was no continuation of this treatment in the post-hatch phase. For *A. boreas*, we ran 42 control replicates, 28 East strain replicates, and 26 West strain that contained a total of 384 surviving hatchlings for a total of 96 experimental units. For *L. catesbeianus*, we ran 23 control replicates, 17 East strain replicates, and 16 West strain using a total of 228 surviving hatchlings with a total of 56 experimental units. Due to complete mortality in the East/Early and the West/Early pre-hatch phase, these treatments were not continued in the post-hatch phase (Table 2).

**Table 2:** Number of replicated groups exposed in the different treatments per species as larvae.

		**Pre-hatching treatments**					
		***Bd*** **strain** **×** **Time**					
		**Control**		**East**		**West**	
		**Early**	**Late**	**Early**	**Late**	**Early**	**Late**
*Pseudacris regilla*							
Post-hatch	Control	8 (32)	7 (28)	5 (20)	0 (0)	7 (28)	6 (24)
*Bd* treatment	East	8 (32)	7 (28)	6 (24)	0 (0)	–	–
	West	8 (32)	7 (28)	–	–	7 (28)	6 (24)
*Anaxyrus boreas*							
Post-hatch	Control	5 (20)	3 (12)	8 (32)	8 (32)	9 (36)	9 (36)
*Bd* treatment	East	6 (24)	5 (20)	9 (36)	8 (32)	–	–
	West	5 (20)	4 (16)	–	–	9 (36)	8 (32)
*Lithobates catesbeianus*							
Post-hatch	Control	6 (24)	6 (24)	0 (0)	7 (28)	0 (0)	4 (16)
*Bd* treatment	East	5 (20)	5 (20)	0 (0)	7 (28)	–	–
	West	6 (24)	6 (24)	–	–	0 (0)	4 (16)

*In parentheses total number of individuals per treatment including all replicates; (–) no treatment*.

Larvae were held individually and those that were re-exposed to *Bd* were re-inoculated once a week (every 7 d) for the duration of the experiment. Individuals were held in rectangular plastic containers (31 × 18 × 8 cm) filled with 2,000 ml dechlorinated water. Water changes occurred concurrently with re-inoculation using 5 ml of 50 K zoospores/ml. Animals that died during the experiment were preserved in 95% ethanol. At the end of the experiment, animals remaining alive were humanely euthanized in accordance with institutional animal care protocol in MS-222 (Tricaine methanesulfonate) and then preserved in 95% ethanol. The experimental trials for each species lasted until individuals reached Gosner stage 30–31 (distinctive rear limb bud) or death. Total duration for the experiment was 65 days for *P. regilla*, 59 days for *A. boreas* and 19 days for *L. catesbeianus*.

We monitored survival twice per day and quantified developmental differences through time by staging all larvae (Gosner stage) every week during water changes. At the end of the post-hatch phase, we sampled a subset of all *Bd*-exposed animals of each species and also randomly sampled 5 control animals of each species to confirm no contamination happened. To assess infection load at the termination of the experiment, we dissected larvae mouthparts for *P. regilla* individuals, and we swabbed mouthparts using fine tipped sterile rayon swabs (Medical Wire and Equipment MW&E 113) for *A. boreas and L. catesbeianus*. Both protocols, swabbing and cutting mouthparts, are recommended as adequate protocols for assessing infection loads. Excised mouthparts and swabbing are similar in the likelihood of detecting *Bd* infection regardless of developmental stage and larval size (62–64).

Each sample was analyzed using quantitative polymerase chain reaction (qPCR) following the methods of (65). A small modification of the amount of Prepman Ultra (Applied Biosystems^®^, Life Technologies) was used to extract the DNA; we used 60 μL instead of 40 μL (66). Our extractions were diluted 1:10 and each sample was analyzed in triplicate to quantify the average number of genome equivalents per animal (7,500 real-time PCR Applied Biosystems instrument). To analyze infection loads, we log transformed the qPCR results as log (genome equivalents per individual + 1) to normalize data.

Effect of exposure on survivorship was analyzed independently by species using odds ratios calculated with a generalized linear mixed model, family: binomial (logit). The values of the ratios represent the likelihood or the risk of mortality due to exposure to the pathogen in comparison to the controls. Therefore, odds ratios higher than 1 represent an increased risk after exposure, odds ratios equal to one represent no difference in the risk, and odds ratios lower than 1 represent a lower risk of the exposed group. All analyses were run in R (version 4.0.3).

## Results

### Pre-hatching Phase

**Figure 2:**
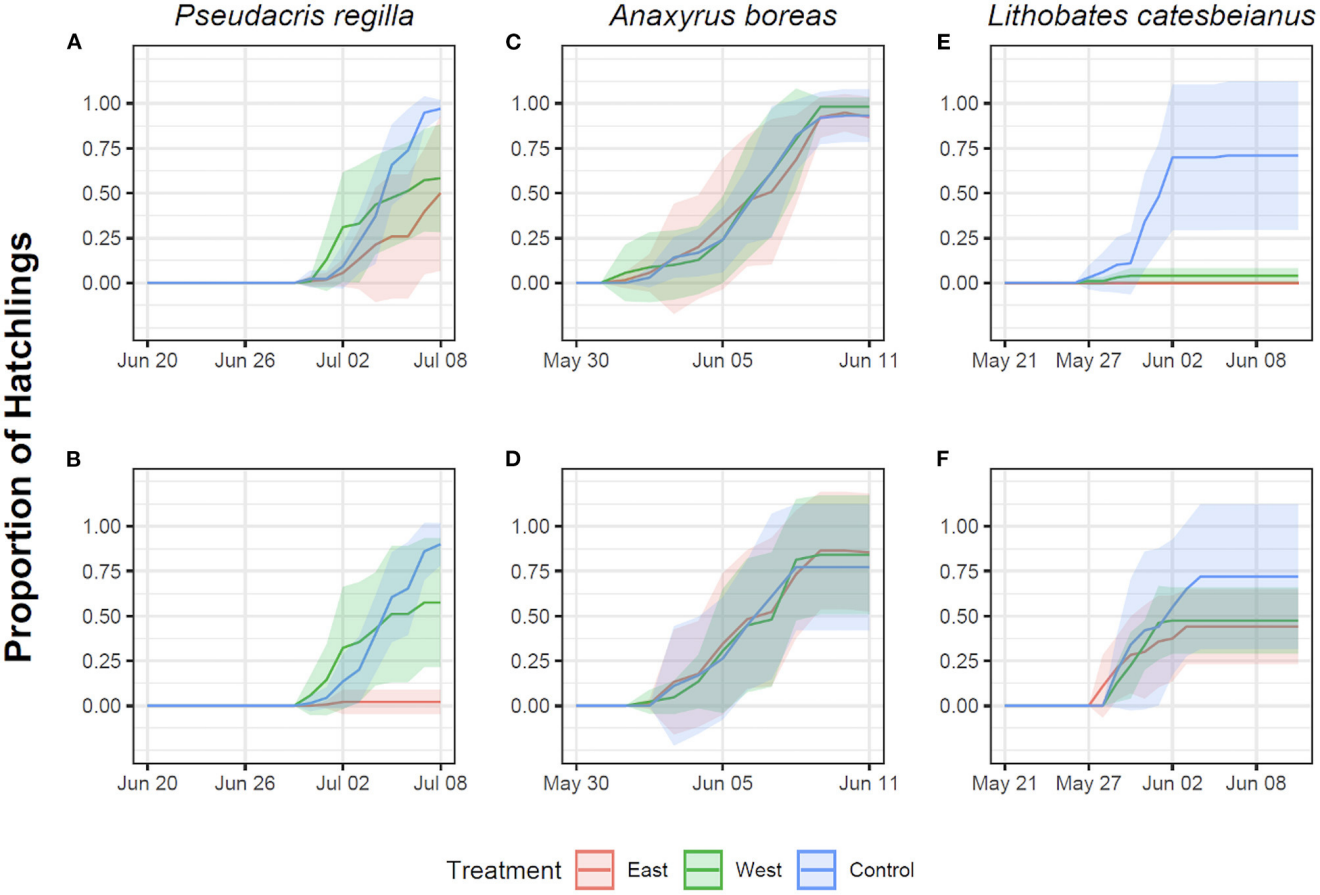
Proportion of hatchlings in *Pseudacris regilla, Anaxyrus boreas*, and *Lithobates catesbeianus* after exposure of eggs to different chytrid treatments. Red represents exposure to the East strain, green represents exposure to the West strain, and controls are indicated in blue. First row represents Early exposure (Gosner stage 12) for *Pseudacris regilla* **(A)**, *Anaxyrus boreas* **(C)** and *Lithobates catesbeianus* **(E)**. Second row represents Late exposure (Gosner stage 18) for *Pseudacris regilla* **(B)**, *Anaxyrus boreas* **(D)** and *Lithobates catesbeianus* **(F)**.

*Pseudacris regilla* embryos exposed to both the East and West *Bd* strains in the Early exposure groups had a lower proportion of hatchlings relative to controls (East strain: *t* = −4.40, *p* < 0.001; West strain: *t* = 1.99, *p* = 0.04). A *post hoc* Tukey test showed that this proportion was different in embryos exposed to the East strain in contrast to the control (*z* = −4.45, *p* < 0.001) and the West strain (*z* = 6.14, *p* < 0.001), with approximately a 50% hatching rate (Figure 2A). Reduced hatching was also found in the Late/East treatment group (*t* = −11.03, *p* < 0.001) relative to the Late/West treatment (*t* = −1.29, *p* = 0.19). In fact, <10% of embryos hatched after being exposed late in development to the non-native East strain (Figure 2B). In *A. boreas*, the proportion of embryos that hatched was similar across both strains in comparison to controls across the Early (East strain: *t* = –0.49, *p* = 0.62; West strain: *t* = 0.62, *p* = 0.53) (Figure 2C) and Late exposure treatments (East strain: *t* = 1.31, *p* = 0.19; West strain: *t* = 0.73, *p* = 0.46) (Figure 2D).

The proportion of *L. catesbeianus* embryos that hatched was low when embryos were exposed early in development, with lower survival in the West *Bd* strain treatment relative to controls (West strain: *t* = 3.58, *p* < 0.001) (Figure 2E). There were no survivors in the East strain exposure treatment. The estimate of *Bd* strain as a factor in our model was high (5,329), potentially due to the 100% mortality, making the *t* and *p*-value not significant (*t* = 0.003, *p* = 0.99). The proportion of embryos that hatched in the Late exposure treatment was lower across both *Bd* strains in comparison to the controls (East strain: *t* = 2.89, *p* < 0.01; West strain: *t* = 2.13, *p* = 0.03) (Figure 2F). A *post hoc* Tukey test showed that this proportion was different in embryos exposed to the East strain in contrast to the control (*z* = 2.89, *p* < 0.01), but it was not different for embryos exposed to the West strain (*z* = 2.13, *p* = 0.08).

### Post-hatching Phase

Our generalized linear mixed model quantified as odds ratios (OR) the effects of exposure to a particular strain on larvae mortality in comparison to the controls given their history of exposure as embryos. As such, results are reported as an increase or decrease in odds of mortality.

### Direct Effects on Larvae -Only Exposed to *Bd* as Larvae (Control-*Bd*)

**Figure 3:**
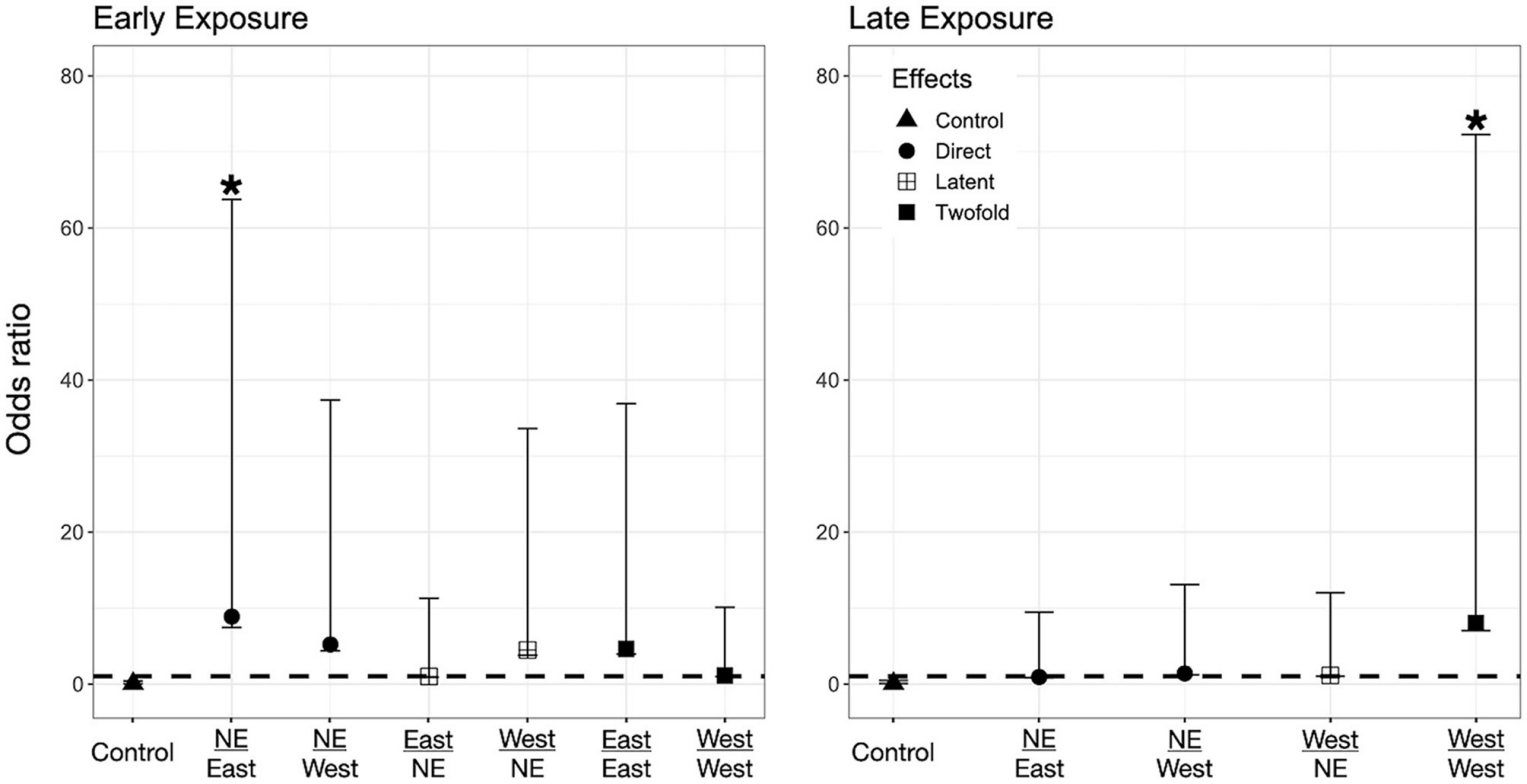
Odds ratio (OR) for *P. regilla* tadpoles according to their exposure regimes. Label of x-axis includes a fraction that indicates in the numerator the exposure regime as embryo and in the denominator exposure regime post-hatching (no exposure = NE, East or West for *Bd* chytrid strain). A dashed line indicates value 1. OR > 1 higher risk after exposure, OR = 1 no risk difference, OR < 1 lower risk after exposure. A star (*) indicates treatments with significant odds ratios. Effects are represented as follows: Direct effects –only exposed to *Bd* as larvae– are represented by circles, latent effects –only exposed as embryos– are represented as four squares, and 2-fold effects –exposed as embryos and larvae– are represented with filled squares; controls are represented by triangles.

**Figure 4:**
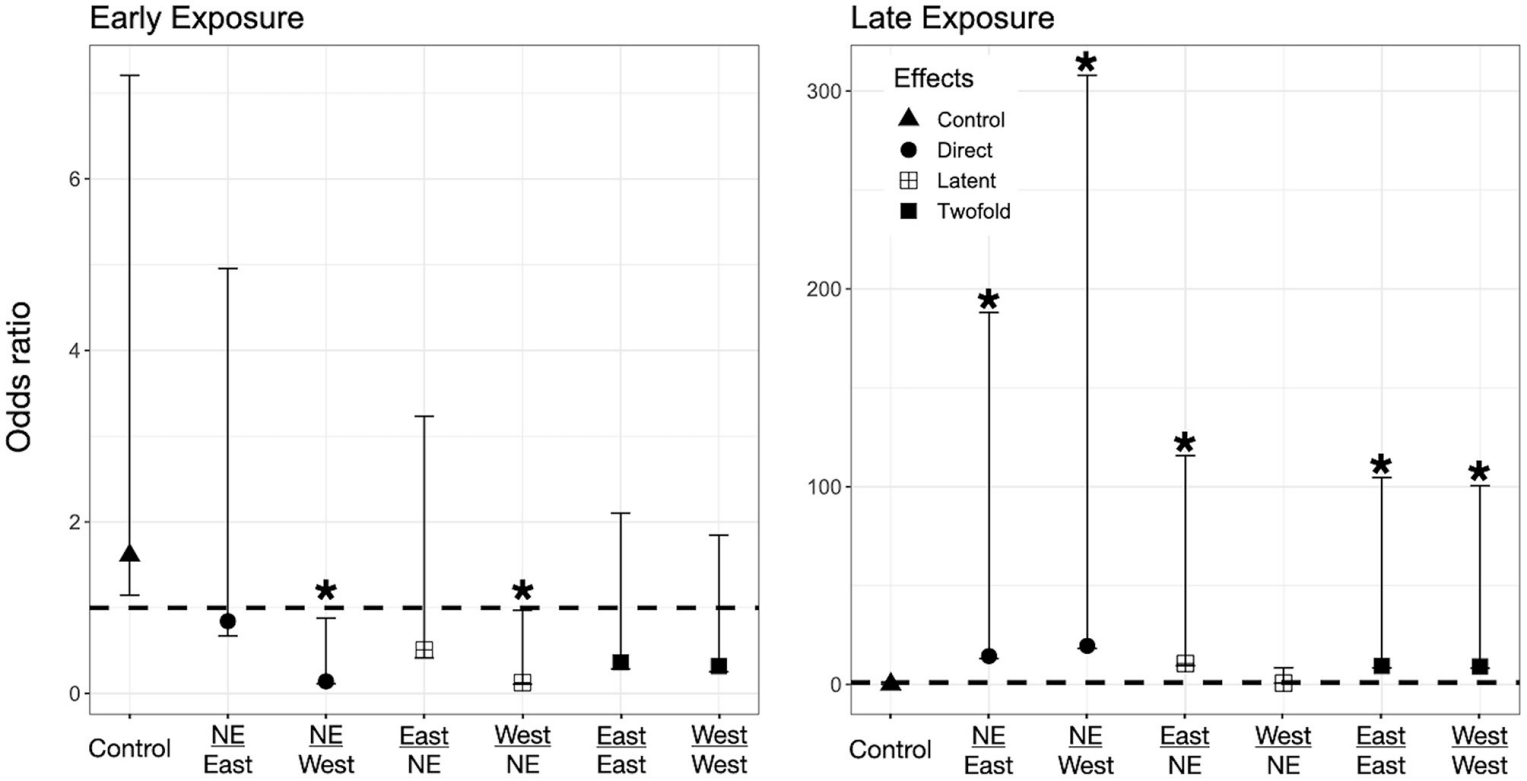
Odds ratio (OR) for *A. boreas* tadpoles according to their exposure regimes. Label of x-axis includes a fraction that indicates in the numerator the exposure regime as embryo and in the denominator exposure regime post-hatching (no exposure = NE, East or West for *Bd* chytrid strain). A dashed line indicates value 1. OR > 1 higher risk after exposure, OR = 1 no risk difference, OR < 1 lower risk after exposure. A star (*) indicates treatments with significant odds ratios. Effects are represented as follow: Direct effects –only exposed to *Bd* as larvae– are represented by circles, latent effects –only exposed as embryos– are represented as four squares, and 2-fold effects –exposed as embryos and larvae– are represented with filled squares; controls are represented by triangles.

**Figure 5:**
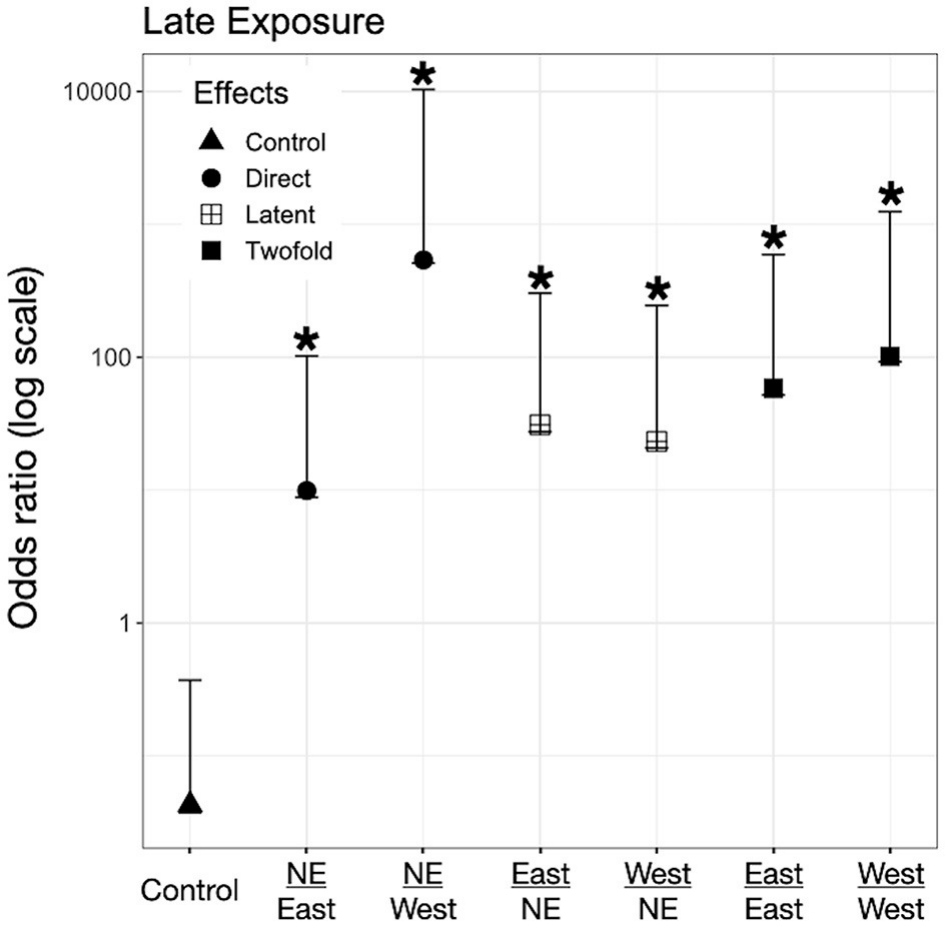
Odds ratio (OR) for *L. catesbeianus* tadpoles according to their exposure regimes. Label of x-axis includes a fraction that indicates in the numerator the exposure regime as embryo and in the denominator exposure regime post-hatching. A star (*) indicates treatments with significant odds ratios. Effects are represented as follow: Direct effects –only exposed to *Bd* as larvae– are represented by circles, latent effects –only exposed as embryos– are represented as four squares, and 2-fold effects –exposed as embryos and larvae– are represented with filled squares; controls are represented by triangles.

We found evidence for direct effects of *Bd* exposure on larval mortality for the three species. In *P. regilla*, post-hatching exposure to the East strain increased the odds of mortality (OR _Early/Control−East_ 8.88, *p* = 0.01, CI. 1.43–54.85, Figure 3, Left panel). For *A. boreas*, we found that individuals exposed during the post-hatch phase to the West strain had lower odds of mortality relative to controls (OR _Early/Control−West_ 0.12, *p* = 0.03, CI: 0.018–0.84, Figure 4, Left panel). In contrast, larvae coming from the Late control group and exposed post-hatch to East or West had higher odds of mortality than controls (OR _Late/Control−East_ 14.38, *p* = 0.03, CI: 1.19–173.65; OR _Late/Control−West_ 19.56, *p* = 0.03, CI: 1.32–288, Figure 4, Right panel). Larvae of *L. catesbeianus* increased their odds of mortality when exposed to either East or West strain (OR _East_ 9.9, *p* = 0.04, CI: 1.06–92; OR _West_ 539, *p* < 0.001, CI: 29.64–9,801) (Figure 5).

### Latent Effects on Larvae -Only Exposed to *Bd* as Embryos (*Bd*-Control)

We did not find evidence for latent effects in *P. regilla*. In *A. boreas* odds of mortality changed according to the time of exposure and *Bd* strain. Odds of mortality for larvae decreased when embryos were exposed early in development to the West strain of *Bd* (OR _Early/West−control_ 0.14, *p* = 0.02, CI: 0.026–0.73, Figure 4, Left panel). On the contrary, individuals exposed Late as embryos to the East strain had higher odds of mortality than controls (OR _Late/East−control_ 10.62, *p* = 0.04, CI: 1.07–105, Figure 4, Right panel). In *L. catesbeianus*, we found higher odds of mortality than controls for both *Bd* strains (OR _Late/East−control_ 31, *p* = 0.001, CI: 3.5–272, OR _Late/West−control_ 23.21, *p* = 0.006, CI: 2.42–222.14, Figure 5).

### Double Exposed Treatments -Exposed to *Bd* as Both Embryos and Larvae (*Bd*-*Bd*)

We found evidence that exposure to *Bd* in both the embryonic and larval stages affect the larval odds of mortality in all three species. We found *P. regilla* that were re-exposed to the West strain, (Late exposure group) increased the odds of mortality (OR _Late/West−West_ 8.05, *p* = 0.04, CI 1.01–64.22, Figure 3, Right panel). In *A. boreas* odds of mortality increased in re-exposed individuals to both the East and West strains (Late exposure groups) (OR _Late/East−East_ 9.37, *p* = 0.05, CI: 0.92–95; OR _Late/West−West_ 9.12, *p* = 0.05, CI: 0.91–91.32) (Figure 4, Right panel). In *L. catesbeianus*, odds of mortality were high for re-exposed animals to either *Bd* strain (OR _East/Late−East_ 58.3, *p* = 0.0003, CI: 6.35–534.6, OR _West/Late−West_ 101.29, *p* < 0.001, CI: 8.9–1,145) (Figure 5).

### Infection Loads

We confirmed *Bd* infection using real-time PCR analyses of tadpole mouthparts for *P. regilla* and swabs for *A. boreas* and *L. catesbeianus*. All tadpoles from control treatments tested negative for *Bd*. Individuals from the three species only exposed as larvae (direct effects) were positive for the East strain. From individuals only exposed as embryos (latent effects), we found that A. *boreas* tested positive for *Bd* when exposed late as embryos with the East strain. In *L. catesbeianus*, individuals tested positive when exposed late to either of the strains (Table 3). In the group of re-exposed individuals (2-fold effects), *P. regilla* was reported *Bd* positive for both East and West strain when exposed early as embryos. In *A. boreas*, individuals were positive for *Bd* when exposed early to the West strain and late when exposed to the East strain (Table 3).

**Table 3:** Mean *Bd* loads (genome equivalents ± SD) at the end of experiment for *P. regilla, A. boreas* and *L. catesbeianus* exposed early (Gosner developmental stage 12) and late (Gosner developmental stage 18) during embryonic development.

		**Direct effects**		**Latent effects**		**2-fold effects**	
**Species**	**Exposure timing**	**CONTROL**		**EAST**	**WEST**	**EAST**	**WEST**
		**East**	**West**	**Control**		**East**	**West**
*P. regilla*	Early	1.25^*^±1.009	1.86 ± 1.24	0	0	0.005 ± 0.009	1.45 ± 1.77
	Late	0.74 ± 1.24	3.39 ± 1.05	NA	0	NA	1.62^*^± 1.17
*A. boreas*	Early	0.008 ± 0.008	0^*^	0	0^*^	0	0.012 ± 0.02
	Late	0.019^*^± 0.01	0^*^	0.00016^*^± 0.0003	0	0.005^*^± 0.006	0
*L. catesbeianus*	Early	0.004 ± 0.006	0.022 ± 0.022	NA	NA	NA	NA
	Late	0.53^*^± 0.62	0	0.03^**^± 0.05	0.59^**^± 0.76	0	0

A star ^*^ indicates significant effects in the odds of mortality of larvae. NA indicates treatments without samples due to mortality in the pre-hatching exposure part.

## Discussion

Life stage, time of exposure, and *Bd* strain influenced susceptibility to *Bd* in the embryo-larvae life history transition for three anuran species: *P. regilla, A. boreas*, and *L. catesbeianus*. We detected direct effects of *Bd* on embryonic and larval mortality, latent effects across the embryo/larval transition, and additive effects when double-exposed to *Bd* across both life stages. Exposure of embryos to *Bd* resulted in direct impacts on hatchling survivorship. We found direct, negative impacts of *Bd* strain and time of exposure on embryonic survival and proportion of hatching success for *P. regilla* and *L. catesbeianus*. Embryos of *P. regilla* were drastically affected by the non-native East *Bd* strain, resulting in more than 90% mortality when exposed later in embryonic development. Interestingly, embryos of invasive *L. catesbeianus* died when exposed to either *Bd* strain (East or West). When exposed early in embryonic development to the East strain, the number of viable hatchlings was zero and we detected a mortality of 90% in hatchlings after early exposure to the West *Bd* strain. When exposed later in development (East or West strains), only 50% of embryos hatched. The influence of time of exposure may be explained by changes in the thickness of the jelly layer surrounding the embryo through development. This jelly layer becomes thinner over development, thus late-stage embryos may be more affected by exposure to a pathogen (15, 18). Amphibian species-specific egg deposition forms (films, strings, clusters), as well as morphology of egg structure, provide differential protection from pathogens (67).

Post-hatching exposure resulted in both direct and latent impacts on larval survivorship. Direct effects on larvae are reported mainly as an increase in the odds of mortality for all three anuran species. *P. regilla* was negatively affected by exposure to the non-native East *Bd* strain, while *A. boreas* and *L. catesbeianus* were affected by both strains (East and West). Odds of mortality in *A. boreas* were higher when exposed to the West strain (19.56) than when exposed to the East strain (14.38). On the contrary, the odds of mortality in *L. catesbeianus* were higher when exposed to the East strain (9.9) than when exposed to the West strain (5.39). This result was not wholly unexpected as larvae mortality has been reported in experimental studies exposing these same species to *Bd*. *A. boreas* has been identified as particularly susceptible to *Bd* (30, 46) while *P. regilla* and *L*. *catesbeianus* larvae have relatively high survivorship (30, 33, 46, 68). In this study, we found a direct effect of *Bd* on larval survivorship for all three species. The increase in the odds of mortality in *P. regilla* and *L. catesbeianus* larvae can be explained by the origin and characteristics of the East strain. Isolated from *L. catesbeianus* in Maine (USA), this strain has been identified as hypervirulent (57, 69, 70) and categorized as part of the North American clade in the Global Pandemic Lineage (GPL) (57). As such, we anticipated an increase in larval mortality due to a lack of evolutionary relationship with this strain. However, *L. catesbeianus* larvae were also susceptible to the East strain even though it was isolated from their conspecifics within their native range.

We detected an increase in the odds of mortality in *P. regilla* when exposed early in embryonic development to *Bd* while the odds ratio for larval mortality were lower for later exposure. We could not evaluate potential latent effects after late exposure of *P. regilla* embryos to the East strain. Effects of *Bd* differed in embryos according to the timing of exposure. However, these effects of timing of exposure and duration of stressors such as pathogens and its relation to critical window and disease is not broadly tested (71, 72). We found an increase in the odds of larval mortality of both *A. boreas* and *L. catesbeianus* as a function of *Bd* strain and timing of embryonic exposure. In *A. boreas*, embryos exposed early to the West strain showed a decrease in the odds of mortality. Conversely, when *A. boreas* were exposed to the East strain late in embryonic development, larvae were almost 10 times more likely to die than control individuals. There was a similar increase in the odds of larval mortality in *L. catesbeianus* when exposed as embryos to any of the *Bd* strains. The high mortality rates in *L. catesbeianus* when exposed early to *Bd* prevented us from understanding potential latent effects for this invasive species. Our results support the hypothesis that timing of pathogen exposure is a major factor that influences host survivorship. Exposures of amphibian embryos to stressors such as pathogens at early stages of development can trigger effects over ontogeny (73). This exposure can later modify other characteristics, such as growth rates (74), mortality rates (75), mass (5), and development of immune response (76).

We also found effects of double *Bd* exposure (exposed in both the embryonic and larval stages) in all three anuran species. All species showed an increase in the odds of larval mortality when the first *Bd* exposure occurred at a later embryonic developmental stage (Gosner stage 18). In *P. regilla*, odds of mortality increased after double exposure to the West strain. Exposure to either strain (East or West) increased the odds of mortality in *A. boreas* and *L. catesbeianus*. Double exposure effects have been reported in experiments examining the larval/metamorph transition (77), thus our experiment provides additional information concerning other life history transitions providing a comprehensive view of the jeopardy through life.

The differential response of *A. boreas* to Early/Late and East/West *Bd* treatments may be explained by the presence of a potential critical window of vulnerability for this species and by the virulence of the *Bd* strain. Late exposure of *A. boreas* embryos to the East strain increased the odds of larval mortality of this species. Fernandez-Beneitez et al. (18) found that embryos of toads (*Bufo calamita* and *Pelobates cultripes*) exposed to *Saprolegnia* spp. at an early developmental stage (Gosner stage 12) suffered no increase in mortality, while embryos challenged at later stages of embryonic development (Gosner stages 15 and 19) were sensitive to the pathogen dying 72 h after exposure. Understanding which species experience latent effects will help target management efforts by identifying how exposure in particular life history stages can change host response. If a species is identified as being particularly susceptible to exposure to *Bd* as embryos, actions such as ex *situ* protection could be useful for its conservation.

Our findings complement the information on susceptibility of *P. regilla* to *Bd* as larvae of this species had previously been reported to be tolerant to certain *Bd* strains (30, 46). Interestingly, we found that this tolerance can change with an individual's previous exposure regime to non-native strains. Our experimental evaluation revealed that *Bd* strains from an invasive species can have harmful consequences on native and even invasive conspecific hosts. Our findings for *A. boreas* support previous work showing species as being susceptible to both the East and West strains of *Bd* (30, 46, 47). In *L. catesbeianus*, larvae and adults have been reported as able to withstand infection loads of the chytrid in different regions (78) and this species is suggested as an asymptomatic carrier or reservoir of *Bd* (79, 80). Our results indicate that larvae can also be susceptible to *Bd* but this response will be mediated by previous exposure in an early life stage. Individuals that were re-exposed to *Bd* were about 50 times more likely to die than individuals kept as controls. This contrasts with previous experimental studies reporting this species as a carrier of *Bd* (30, 32, 60). Generally, those studies directly exposed individuals in the larval stage [Gosner stage (26–30)] without considering previous exposure regimes. In our study, *L. catesbeianus* were vulnerable to *Bd* exposure in response to direct exposure and across life history transitions.

We found species-specific embryonic mortality after exposure to *Bd*. Many pathogens impact anuran embryos, including ranavirus (76), oomycetes (81–84), filamentous ascomycetes (85) and microsporidia (86). The mechanisms of protection offered by the vitelline membrane against ranavirus are unknown. However, bacteriostatic activity of the egg membrane or the capsule (87) or just its role as structural barrier when exposed to contaminants have been proposed (88, 89). In the case of Oomycetes, zoospores are chemotactic and move toward suitable substrates where they can germinate and grow. Similarly, ascomycetes are able to grow through the jelly. Few studies have suggested how *Bd* affects anuran embryos. *Bd* enzymatic action is one mechanism that could explain this result, as it can cause damage in skin tissue of hosts after exposure (30, 49, 90, 91). A complex mix of proteolytic and hydrolyzing enzymes (esterases) that degrade amphibian tissue have been described from different *Bd* isolates (90–92). In addition, many hatching anurans release enzymes to assist with degradation of the egg capsule at the moment of hatching (93, 94); this could potentially facilitate the enzymatic action of *Bd* to degrade tissues. Recently, dose-dependent mortality and proliferation in zebrafish (*Danio rerio*) tissue was reported with toxins secreted after the establishment of *Bd* sporangia (95).

The present study offers useful information about the complexity of host response to a pathogen, particularly with multiple exposures across life stages. Our study provides information about direct effects of *Bd* on anuran embryos, with significant impacts on mortality and the proportion of hatching success. Our results also quantified latent effects of *Bd* exposure over ontogeny (96). Despite being a relatively brief period, exposure to *Bd* in the egg led to increased mortality after hatching and species-specific differences were due to the timing of embryonic exposure and re-exposure in the larval stage. As we increase our understanding of how *Bd* impacts amphibians through direct and even latent effects, we are recognizing that the effects *Bd* may have on population dynamics and conservation of amphibians have been underestimated in wild populations.

Additional research exploring the mechanisms protecting the embryos is needed to better understand the susceptibility of this developmental stage to disease. Characteristics such as jelly thickness and composition, or size of the capsule, can be involved in resistance to chytrid. As eggs received material from their parents during oviposition, evaluating the role of parents in the immune response of their offspring can help us to understand more about embryonic immunity. As continued survey efforts have located *Bd* in populations of amphibians around the world, there is growing evidence that the risk of *Bd* cannot be simplified to species susceptibility. Instead, *Bd* risk includes strain, host life-stage, and specific exposure scenarios. Further studies are also required to better understand how variation in other environmental and biological parameters can affect the outcome of repeated *Bd* exposure in anuran species. Our results add information to the growing body of evidence concerning differential susceptibility to pathogens among amphibian species and across life stages.

## Data Availability Statement

The raw data supporting the conclusions of this article will be made available by the authors, without undue reservation.

## Ethics Statement

The animal study was reviewed and approved by Institutional Animal Care and Use committee Oregon State University.

## Author Contributions

JU, AB, and TG conceptualized the manuscript. JU and EB performed the field work, experiments and data analysis. JU, EB, AB, and TG have made a significant, direct and intellectual contribution to the work. All authors contributed to the article and approved the submitted version.

## Funding

Publication of this paper was supported, in part, by the Henry Mastin Graduate Student Fund (Department of Fisheries, Wildlife and Conservation Sciences, Oregon State University) and the US Department of Agriculture, Forest Service, Pacific Northwest Research Station through Deanna H. Olson.

## Conflict of Interest

The authors declare that the research was conducted in the absence of any commercial or financial relationships that could be construed as a potential conflict of interest.

## Publisher's Note

All claims expressed in this article are solely those of the authors and do not necessarily represent those of their affiliated organizations, or those of the publisher, the editors and the reviewers. Any product that may be evaluated in this article, or claim that may be made by its manufacturer, is not guaranteed or endorsed by the publisher.

## References

[1] Merilä J Svensson E. Are fat reserves in migratory birds affected by condition in early life? J Avian Biol. (1997) 28:279–86. 10.2307/3676940

[2] Miller K Packard GC Packard MJ. Hydric conditions during incubation influence locomotor performance of hatchling snapping turtles. J Exp Biol. (1987) 127:401–12. 10.1242/jeb.127.1.401

[3] Heintz RA Rice SD Wertheimer AC Bradshaw RF Thrower FP Joyce JE. Delayed effects on growth and marine survival of pink salmon *Oncorhynchus gorbuscha* after exposure to crude oil during embryonic development. Mar Ecol Prog Ser. (2000) 208:205–16. 10.3354/meps208205

[4] Pechenik JA. Larval experience and latent effects—metamorphosis is not a new beginning. Integr Comp Biol. (2006) 46:323–33. 10.1093/icb/icj028 21672745

[5] Uller T Sagvik J Olsson M. Pre-hatching exposure to water mold reduces size at metamorphosis in the moor frog. Oecologia. (2009) 160:9–14. 10.1007/s00442-009-1280-6 19189128

[6] Murillo-Rincón AP Laurila A Orizaola G. Compensating for delayed hatching reduces offspring immune response and increases life-history costs. Oikos. (2017) 126:565–71. 10.1111/oik.04014

[7] Sniegula S Janssens L Stoks R. Integrating multiple stressors across life stages and latitudes: combined and delayed effects of an egg heat wave and larval pesticide exposure in a damselfly. Aquatic Toxicology. (2017) 186:113–22. 10.1016/j.aquatox.2017.02.029 28282618

[8] LaFiandra EM Babbitt KJ. Predator induced phenotypic plasticity in the pinewoods tree frog, *Hyla femoralis*: necessary cues and the cost of development. Oecologia. (2004) 138:350–9. 10.1007/s00442-003-1412-3 14673637

[9] Richter-Boix A Orizaola G Laurila A. Transgenerational phenotypic plasticity links breeding phenology with offspring life-history. Ecology. (2014) 95:2715–22. 10.1890/13-1996.1

[10] Garcia TS Urbina J Bredeweg E Ferrari MCO. Embryonic learning and developmental carry-over effects in an invasive anuran. Oecologia. (2017) 184:623–31 10.1007/s00442-017-3905-5 28669002

[11] Sih A Moore RD. Delayed hatching of salamander eggs in response to enhanced larval predation risk. Am Nat. (1993) 142:947–60. 10.1086/285583 19425943

[12] Warkentin KM. Adaptive plasticity in hatching age: a response to predation risk trade-offs. Proc Nat Acad Sci. (1995) 92:3507–10. 10.1073/pnas.92.8.3507 11607529 PMC42196

[13] Warkentin KM. Plasticity of hatching in amphibians: evolution, trade-offs, cues and mechanisms. Integr Comp Biol. (2011) 51:111–27. 10.1093/icb/icr046 21642239

[14] Chivers DP Kiesecker JM Marco A Devito J Anderson MT Blaustein AR. Predator-induced life history changes in amphibians: egg predation induces hatching. Oikos. (2001) 92:135–42. 10.1034/j.1600-0706.2001.920116.x

[15] Gomez-Mestre I Touchon JC Warkentin KM. Amphibian embryo and parental defenses and a larval predator reduce egg mortality from water mold. Ecology. (2006) 87:2570–81. 10.1890/0012-9658(2006)87[2570:AEAPDA]2.0.CO;2 17089665

[16] Touchon JTJ Gomez-Mestre IG-MI Warkentin KWK. Hatching plasticity in two temperate anurans: responses to a pathogen and predation cues. Can J Zool. (2006) 84:556–63. 10.1139/z06-058

[17] Touchon JC McCoy MW Vonesh JR Warkentin KM. Effects of plastic hatching timing carry over through metamorphosis in red-eyed treefrogs. Ecology. (2013) 94:850–60. 10.1890/12-0194.1

[18] Fernández-Benéitez M Ortiz-Santaliestra M Lizana M Diéguez-Uribeondo J. Differences in susceptibility to Saprolegnia infections among embryonic stages of two anuran species. Oecologia. (2011) 165:819–26. 10.1007/s00442-010-1889-5 21197546

[19] Rumschlag SL Boone MD. How time of exposure to the amphibian chytrid fungus affects *Hyla chrysoscelis* in the presence of an insecticide. Herpetologica. (2015) 71:169–76. 10.1655/HERPETOLOGICA-D-13-00070

[20] Develey-Rivière M-P Galiana E. Resistance to pathogens and host developmental stage: a multifaceted relationship within the plant kingdom. New Phytol. (2007) 175:405–16. 10.1111/j.1469-8137.2007.02130.x 17635216

[21] Brutscher LM Daughenbaugh KF Flenniken ML. Antiviral defense mechanisms in honey bees. Curr Opin Insect Sci. (2015) 10:71–82. 10.1016/j.cois.2015.04.016 26273564 PMC4530548

[22] Mast J Goddeeris BM. Development of immunocompetence of broiler chickens. Vet Immunol Immunopathol. (1999) 70:245–56. 10.1016/S0165-2427(99)00079-3 10507364

[23] Holgersson MCN Nichols WA Paitz RT Bowden RM. How important is the eggshell as a source for initial acquisition of Salmonella in hatchling turtles? J Exp Zool. (2016) 325:142–8. 10.1002/jez.2004 26817746

[24] Valkenburg SA Venturi V Dang THY Bird NL Doherty PC Turner SJ. Early priming minimizes the age-related immune compromise of CD8^+^ T cell diversity and function. PLoS Pathog. (2012) 8:e1002544. 10.1371/journal.ppat.1002544 22383879 PMC3285595

[25] Rohr JR Raffel TR Hall CA. Developmental variation in resistance and tolerance in a multi-host–parasite system. Functional Ecology. (2010) 24:1110–21. 10.1111/j.1365-2435.2010.01709.x

[26] Echaubard P Pauli BD Trudeau VL Lesbarrères D. Ranavirus infection in northern leopard frogs: the timing and number of exposures matter. J Zool. (2016) 298:30–6. 10.1111/jzo.12281

[27] Hatcher MJ Dick JTA Dunn AM. Disease emergence and invasions. Funct Ecol. (2012) 26:1275–87. 10.1111/j.1365-2435.2012.02031.x 32313353 PMC7163950

[28] Olson DH Aanensen DM Ronnenberg KL Powell CI Walker SF Bielby J. The *Bd* mapping group. Mapping the global emergence of *Batrachochytrium dendrobatidis*, the amphibian chytrid fungus. PLoS ONE. (2013) 8:e56802. 10.1371/journal.pone.0056802 23463502 PMC3584086

[29] Berger L Roberts AA Voyles J Longcore JE Murray KA Skerratt LF. History and recent progress on chytridiomycosis in amphibians. Fungal Ecol. (2016) 19:89–99. 10.1016/j.funeco.2015.09.007

[30] Blaustein AR Romansic JM Scheessele EA Han BA Pessier AP Longcore JE. Interspecific variation in susceptibility of frog tadpoles to the pathogenic fungus *Batrachochytrium dendrobatidis*. Conservation Biology. (2005) 19:1460–8. 10.1111/j.1523-1739.2005.00195.x

[31] Searle CL Gervasi SS Hua J Hammond JI Relyea RA Olson DH. Differential host susceptibility to *Batrachochytrium dendrobatidis*, an emerging amphibian pathogen. Conservation Biology. (2011) 25:965–74. 10.1111/j.1523-1739.2011.01708.x 21732979

[32] Gahl MK Longcore JE Houlahan JE. Varying responses of northeastern north american amphibians to the chytrid pathogen *Batrachochytrium dendrobatidis*. Conservation Biology. (2012) 26:135–41. 10.1111/j.1523-1739.2011.01801.x 22181933

[33] Gervasi S Gondhalekar C Olson DH Blaustein AR. Host identity matters in the amphibian-*Batrachochytrium dendrobatidis* system: fine-scale patterns of variation in responses to a multi-host pathogen. PLoS ONE. (2013) 8:e54490. 10.1371/journal.pone.0054490 23382904 PMC3554766

[34] Gervasi SS Stephens PR Hua J Searle CL Xie GY Urbina J. Linking ecology and epidemiology to understand predictors of multi-host responses to an emerging pathogen, the amphibian chytrid fungus. PLoS ONE. (2017) 12:e0167882. 10.1371/journal.pone.0167882 28095428 PMC5240985

[35] Bielby J Fisher MC Clare FC Rosa GM Garner TWJ. Host species vary in infection probability, sub-lethal effects, and costs of immune response when exposed to an amphibian parasite. Sci Rep. (2015) 5:10828. 10.1038/srep10828 26022346 PMC4448222

[36] Tobler U Schmidt BR. Within- and among-population variation in chytridiomycosis-induced mortality in the toad *Alytes obstetricans*. PLoS ONE. (2010) 5:e10927. 10.1371/journal.pone.0010927 20532196 PMC2880007

[37] Bradley PW Gervasi SS Hua J Cothran RD Relyea RA Olson DH. Differences in sensitivity to the fungal pathogen *Batrachochytrium dendrobatidis* among amphibian populations: pathogen effects across populations. Conservation Biology. (2015) 29:1347–56. 10.1111/cobi.12566 26219571

[38] Briggs CJ Vredenburg VT Knapp RA Rachowicz LJ. Investigating the population-level effects of chytridiomycosis: an emerging infectious disease of amphibians. Ecology. (2005) 86:3149–59. 10.1890/04-1428

[39] Briggs CJ Knapp RA Vredenburg VT. Enzootic and epizootic dynamics of the chytrid fungal pathogen of amphibians. Proc Nat Acad Sci. (2010) 107:9695–700. 10.1073/pnas.0912886107 20457916 PMC2906864

[40] Garner TWJ Walker S Bosch J Leech S Marcus Rowcliffe J Cunningham AA. Life history tradeoffs influence mortality associated with the amphibian pathogen *Batrachochytrium dendrobatidis*. Oikos. (2009) 118:783–91. 10.1111/j.1600-0706.2008.17202.x

[41] Piovia-Scott J Pope KL Lawler SP Cole EM Foley JE. Factors related to the distribution and prevalence of the fungal pathogen *Batrachochytrium dendrobatidis* in *Rana cascadae* and other amphibians in the Klamath Mountains. Biol Conserv. (2011) 144:2913–21. 10.1016/j.biocon.2011.08.008

[42] Ortiz-Santaliestra ME Rittenhouse TAG Cary TL Karasov WH. Interspecific and postmetamorphic variation in susceptibility of three north american anurans to *Batrachochytrium dendrobatidis*. J Herpetol. (2013) 47:286–92. 10.1670/11-134

[43] Retallick RWR Miera V. Strain differences in the amphibian chytrid *Batrachochytrium dendrobatidis* and non-permanent, sub-lethal effects of infection. Dis Aquat Org. (2007) 75:201–7. 10.3354/dao075201 17629114

[44] Doddington BJ Bosch J Oliver JA Grassly NC Garcia G Schmidt BR. Context-dependent amphibian host population response to an invading pathogen. Ecology. (2013) 94:1795–804. 10.1890/12-1270.1 24015523

[45] Piovia-Scott J Pope K Joy Worth S Rosenblum EB Poorten T Refsnider J. Correlates of virulence in a frog-killing fungal pathogen: evidence from a California amphibian decline. ISME J. (2015) 9:1570–8. 10.1038/ismej.2014.241 25514536 PMC4478697

[46] Dang T Searle CL Blaustein AR. Virulence variation among strains of the emerging infectious fungus *Batrachochytrium dendrobatidis* (Bd) in multiple amphibian host species. Dis Aquat Org. (2017) 124:233–9. 10.3354/dao03125 28492179

[47] Gervasi SS Urbina J Hua J Chestnut T Relyea RA Blaustein AR. Experimental evidence for American bullfrog (*Lithobates catesbeianus*) susceptibility to chytrid fungus (*Batrachochytrium dendrobatidis*). Ecohealth. (2013) 10:166–71. 10.1007/s10393-013-0832-8 23539129

[48] Rosenblum EB Fisher MC James TY Stajich JE Longcore JE Gentry LR. Molecular perspective: biology of the emerging pathogen *Batrachochytrium dendrobatidis*. Dis Aquat Organ. (2010) 92:131–47. 10.3354/dao02179 21268975

[49] McMahon TA Brannelly LA Chatfield MWH Johnson PTJ Joseph MB McKenzie VJ. Chytrid fungus *Batrachochytrium dendrobatidis* has nonamphibian hosts and releases chemicals that cause pathology in the absence of infection. Proc Nat Acad Sci. (2013) 110:210–5. 10.1073/pnas.1200592110 23248288 PMC3538220

[50] Fites JS Ramsey JP Holden WM Collier SP Sutherland DM Reinert LK. The invasive chytrid fungus of amphibians paralyzes lymphocyte responses. Science. (2013) 342:366. 10.1126/science.1243316 24136969 PMC3956111

[51] Voyles J Young S Berger L Campbell C Voyles WF Dinudom A. pathogenesis of chytridiomycosis, a cause of catastrophic amphibian declines. Science. (2009) 326:582–5. 10.1126/science.1176765 19900897

[52] Dmitrieva EV. Influence of the concentration of dissolved oxygen on embryonic development of the common toad (*Bufo bufo*). Russ J Dev Biol. (2015) 46:368–80. 10.1134/S1062360415060041 26859970

[53] Blaustein A Beatty J Olson D Storm R. The biology of amphibians and reptiles in old-growth forests in the Pacific Northwest. In: General Technical Report (GTR). Portland, OR: Pacific Northwest Research Station (1995). p.98. 10.2737/PNW-GTR-337

[54] Muths E Corn PS Pessier AP Green DE. Evidence for disease-related amphibian decline in Colorado. Biol Conserv. (2003) 110:357–65. 10.1016/S0006-3207(02)00239-2

[55] Jones LLC Leonard WP Olson DH. Amphibians of the Pacific Northwest. Seattle, WA: Seattle Audubon Society (2005). p. 227

[56] Gosner KL, A. simplified table for staging anuran embryos and larvae with notes on identification. Herpetologica. (1960) 16:183–90.

[57] Schloegel LM Toledo LF Longcore JE Greenspan SE Vieira CA Lee M. Novel, panzootic and hybrid genotypes of amphibian chytridiomycosis associated with the bullfrog trade. Mol Ecol. (2012) 21:5162–77. 10.1111/j.1365-294X.2012.05710.x 22857789

[58] Marcum R St-Hilaire S Murphy P Rodnick K. Effects of *Batrachochytrium dendrobatidis* infection on ion concentrations in the boreal toad *Anaxyrus* (Bufo) *boreas boreas*. Dis Aquat Org. (2010) 91:17–21. 10.3354/dao02235 20853738

[59] Searle CL Belden LK Du P Blaustein AR. Stress and chytridiomycosis: exogenous exposure to corticosterone does not alter amphibian susceptibility to a fungal pathogen. J Exp Zool. (2014) 321:243–53. 10.1002/jez.1855 24610865

[60] Eskew EA Worth SJ Foley JE Todd BD. American bullfrogs (*Lithobates catesbeianus*) resist infection by multiple isolates of *Batrachochytrium dendrobatidis*, including one implicated in wild mass mortality. Ecohealth. (2015) 12:513–8. 10.1007/s10393-015-1035-2 26065669

[61] R Core Team. R: A language and environment for statistical computing. Vienna, Au: R Foundation for Statistical Computing (2016). Available online at: http://www.R-project.org/

[62] Retallick RWR Miera V Richards KL Field KJ Collins JP, A. non-lethal technique for detecting the chytrid fungus *Batrachochytrium dendrobatidis* on tadpoles. Dis Aquat Org. (2006) 72:77–85. 10.3354/dao072077 17067076

[63] Hyatt AD Boyle DG Olsen V Boyle DB Berger L Obendorf D. Diagnostic assays and sampling protocols for the detection of *Batrachochytrium dendrobatidis*. Dis Aquat Organ. (2007) 73:175–92. 10.3354/dao073175 17330737

[64] Kadekaru S Une Y. Comparison of methods for detection of chytrid fungus (*Batrachochytrium dendrobatidis*) in bullfrog tadpole mouthparts. J Vet Med Sci. (2018) 80:260–2. 10.1292/jvms.17-0071 29269708 PMC5836761

[65] Boyle D Boyle D Olsen V Morgan J Hyatt A. Rapid quantitative detection of chytridiomycosis (*Batrachochytrium dendrobatidis*) in amphibian samples using real-time Taqman PCR assay. Dis Aquat Organ. (2004) 60:141–8. 10.3354/dao060141 15460858

[66] Searle CL Xie GY Blaustein AR. Development and infectious disease in hosts with complex life cycles. PLoS ONE. (2013) 8:e60920. 10.1371/journal.pone.0060920 23565288 PMC3615074

[67] Altig R McDiarmid RW. Morphological diversity and evolution of egg and clutch structure in amphibians. Herpetol Monogr. (2007) 21:1–32. 10.1655/06-005.1

[68] Reeder NM Pessier AP Vredenburg VT. A reservoir species for the emerging amphibian pathogen *Batrachochytrium dendrobatidis* thrives in a landscape decimated by disease. PLoS ONE. (2012) 7:e33567. 10.1371/journal.pone.0033567 22428071 PMC3299797

[69] Farrer RA Weinert LA Bielby J Garner TWJ Balloux F Clare F. Multiple emergences of genetically diverse amphibian-infecting chytrids include a globalized hypervirulent recombinant lineage. Proc Nat Acad Sci. (2011) 108:18732–6. 10.1073/pnas.1111915108 22065772 PMC3219125

[70] Rosenblum EB James TY Zamudio KR Poorten TJ Ilut D Rodriguez D. Complex history of the amphibian-killing chytrid fungus revealed with genome resequencing data. Proc Nat Acad Sci. (2013) 110:9385–90. 10.1073/pnas.1300130110 23650365 PMC3677446

[71] Johnson PTJ Kellermanns E Bowerman J. Critical windows of disease risk: amphibian pathology driven by developmental changes in host resistance and tolerance. Funct Ecol. (2011) 25:726–34. 10.1111/j.1365-2435.2010.01830.x

[72] Kirschman LJ Crespi EJ Warne RW. Critical disease windows shaped by stress exposure alter allocation trade-offs between development and immunity. J Anim Ecol. (2018) 87:235–46. 10.1111/1365-2656.12778 29095486

[73] Rohr JR Raffel TR Halstead NT McMahon TA Johnson SA Boughton RK Martin LB. Early-life exposure to a herbicide has enduring effects on pathogen-induced mortality. Proc Biol Soc B. (2013) 280:20131502. 10.1098/rspb.2013.1502 24266041 PMC3813324

[74] Capellán E Nicieza A. Trade-offs across life stages: does predator induced hatching plasticity reduce anuran post-metamorphic performance? Evol Ecol. (2007) 21:445–58. 10.1007/s10682-006-9133-9

[75] Vonesh JR Bolker BM. Compensatory larval responses shift trade-offs associated with predator-induced hatching plasticity. Ecology. (2005) 86:1580–91. 10.1890/04-0535

[76] Haislip NA Gray MJ Hoverman JT Miller DL. Development and disease: how susceptibility to an emerging pathogen changes through anuran development. PLoS ONE. (2011) 6:e22307. 10.1371/journal.pone.0022307 21799820 PMC3142128

[77] Fernández-Loras A Fernández-Beaskoetxea S Arriero E Fisher MC Bosch J. Early exposure to *Batrachochytrium dendrobatidis* causes profound immunosuppression in amphibians. Eur J Wildl Res. (2017) 63:99. 10.1007/s10344-017-1161-y

[78] Hanselmann R Rodriguez A Lampo M Fajardo-Ramos L Aguirre A Kilpatrick AM. Presence of an emerging pathogen of amphibians in introduced bullfrogs *Rana catesbeiana* in Venezuela. Biol Conserv. (2004) 120:115–9. 10.1016/j.biocon.2004.02.013

[79] Daszak P Strieby A Cunningham AA Longcore J Brown C Porter D. Experimental evidence that the bullfrog (*Rana catesbeiana*) is a potential carrier of chytridiomycosis, an emerging fungal disease of amphibians. Herpetol J. (2004) 14:201–7. Available online at: https://www.thebhs.org/publications/the-herpetological-journal/volume-14-number-4-october-2004/1773-06-experimental-evidence-that-the-bullfrog-rana-catesbeiana-is-a-potential-carrier-of-chytridiomycosis-an-emerging-fungal-disease-of-amphibians/file

[80] Garner TWJ Perkins MW Govindarajulu P Seglie D Walker S Cunningham AA. The emerging amphibian pathogen *Batrachochytrium dendrobatidis* globally infects introduced populations of the North American bullfrog, *Rana catesbeiana*. Biol Lett. (2006) 2:455. 10.1098/rsbl.2006.0494 17148429 PMC1686185

[81] Kiesecker JM Blaustein AR. Synergism between UV-B radiation and a pathogen magnifies amphibian embryo mortality in nature. Proc Natl Acad Sci USA. (1995) 92:11049–52. 10.1073/pnas.92.24.11049 7479934 PMC40568

[82] Fernández-Benéitez MJ Ortiz-Santaliestra ME Lizana M Diéguez-Uribeondo J. *Saprolegnia diclina*: another species responsible for the emergent disease ‘Saprolegnia infections’ in amphibians. FEMS Microbiol Lett. (2008) 279:23–9. 10.1111/j.1574-6968.2007.01002.x 18177304

[83] Ruthig GR. The influence of temperature and spatial distribution on the susceptibility of southern leopard frog eggs to disease. Oecologia. (2008) 156:895–903. 10.1007/s00442-008-1026-x 18368423

[84] Ruthig G. Water molds of the genera Saprolegnia and Leptolegnia are pathogenic to the North American frogs *Rana catesbeiana* and *Pseudacris crucifer*, respectively. Dis Aquat Org. (2009) 84:173–8. 10.3354/dao02042 19565694

[85] Warkentin KM Currie CR Rehner SA. Egg-killing fungus induces early hatching of red-eyed treefrog eggs. Ecology. (2001) 82:2860–9. 10.1890/0012-9658(2001)082[2860:EKFIEH]2.0.CO;2

[86] Green DE Converse KA. Diseases of amphibian eggs and embryos. In: Majumdar SK Huffman JE Brenner FJ Panah AI, editors. Wildlife Diseases: Landscape Epidemiology, Spatial Distribution and Utilization of Remote Sensing Technology. Easton, PA: The Pennsylvania Academy of Science (2005). p. 62–71. Available online at: http://pubs.er.usgs.gov/publication/85665

[87] Han Y Yu H Yang X Rees HH Liu J Lai R. serine proteinase inhibitor from frog eggs with bacteriostatic activity. Comp Biochem Physiol B Biochem Mol Biol. (2008) 149:58–62. 10.1016/j.cbpb.2007.08.003 17826205

[88] Berrill M Coulson D McGillivray L Pauli B. Toxicity of endosulfan to aquatic stages of anuran amphibians. Environ Toxicol Chem. (1998) 17:1738–44. 10.1002/etc.5620170914

[89] Pauli BD Coulson DR Berrill M. Sensitivity of amphibian embryos and tadpoles to Mimic^®^ 240 LV insecticide following single or double exposures. Environ Toxicol Chem. (1999) 18:2538–44. 10.1002/etc.5620181122

[90] Symonds EP Trott DJ Bird PS Mills P. Growth characteristics and enzyme activity in *Batrachochytrium dendrobatidis* isolates. Mycopathologia. (2008) 166:143–7. 10.1007/s11046-008-9135-y 18568420

[91] Moss A Carty N San Francisco M. Identification and partial characterization of an elastolytic protease in the amphibian pathogen *Batrachochytrium dendrobatidis*. Dis Aquat Org. (2010) 92:149–58. 10.3354/dao02223 21268976

[92] Brutyn M D'Herde K Dhaenens M Rooij PV Verbrugghe E Hyatt AD. *Batrachochytrium dendrobatidis* zoospore secretions rapidly disturb intercellular junctions in frog skin. Fungal Genet Biol. (2012) 49:830–7. 10.1016/j.fgb.2012.07.002 22903040

[93] Carroll EJ Hedrick JL. Hatching in the toad *Xenopus laevis*: morphological events and evidence for a hatching enzyme. Dev Biol. (1974) 38:1–13. 10.1016/0012-1606(74)90254-1 4826290

[94] Cohen KL Seid MA Warkentin KM. How embryos escape from danger: the mechanism of rapid, plastic hatching in red-eyed treefrogs. J Exp Biol. (2016) 219:1875. 10.1242/jeb.139519 27307544

[95] Liew N Mazon Moya MJ Wierzbicki CJ Hollinshead M Dillon MJ Thornton CR. Chytrid fungus infection in zebrafish demonstrates that the pathogen can parasitize non-amphibian vertebrate hosts. Nat Commun. (2017) 8:15048. 10.1038/ncomms15048 28425465 PMC5411484

[96] Hamdoun A Epel D. Embryo stability and vulnerability in an always changing world. Proc Nat Acad Sci. (2007) 104:1745–50. 10.1073/pnas.0610108104 17264211 PMC1794293

